# Properties and Applications of High Emissivity Composite Films Based on Far-Infrared Ceramic Powder

**DOI:** 10.3390/ma10121370

**Published:** 2017-11-29

**Authors:** Yabo Xiong, Shaoyun Huang, Wenqi Wang, Xinghai Liu, Houbin Li

**Affiliations:** School of Printing and Packaging, Wuhan University, No. 129 Luo Yu Road, Wuchang District, Wuhan 430079, China; xiongyabo1030@whu.edu.cn (Y.X.); xiaoye8612@163.com (S.H.); wwqwhu93@gmail.com (W.W.); liuxh@whu.edu.cn (X.L.)

**Keywords:** ceramic powder, far-infrared emissivity, composite film, strawberries

## Abstract

Polymer matrix composite materials that can emit radiation in the far-infrared region of the spectrum are receiving increasing attention due to their ability to significantly influence biological processes. This study reports on the far-infrared emissivity property of composite films based on far-infrared ceramic powder. X-ray fluorescence spectrometry, Fourier transform infrared spectroscopy, thermogravimetric analysis, and X-ray powder diffractometry were used to evaluate the physical properties of the ceramic powder. The ceramic powder was found to be rich in aluminum oxide, titanium oxide, and silicon oxide, which demonstrate high far-infrared emissivity. In addition, the micromorphology, mechanical performance, dynamic mechanical properties, and far-infrared emissivity of the composite were analyzed to evaluate their suitability for strawberry storage. The mechanical properties of the far-infrared radiation ceramic (cFIR) composite films were not significantly influenced (*p* ≥ 0.05) by the addition of the ceramic powder. However, the dynamic mechanical analysis (DMA) properties of the cFIR composite films, including a reduction in damping and shock absorption performance, were significant influenced by the addition of the ceramic powder. Moreover, the cFIR composite films showed high far-infrared emissivity, which has the capability of prolonging the storage life of strawberries. This research demonstrates that cFIR composite films are promising for future applications.

## 1. Introduction

Polymer composites with high strength, chemical and abrasion resistance, and desirable thermal and mechanical properties are increasingly used to replace metal components because of their improved strength-to-density ratios. For example, nylon, polythene, and polypropylene are commonly used polymer or fiber materials with wide-ranging applications such as in car parts, office materials, and hospital instruments. These materials have low densities, high specific strengths and moduli, and a low cost and are easy to process [1,2]. However, these polymers, which are based on petrochemical compounds, do not degrade easily in the natural environment [3]. To further widen applications of polymer products and reduce environmental pollution, different fillers are added to form polymer composites. Nevertheless, such composite structure can often lead to less desirable mechanical properties.

Recent studies have shown that ceramic composites have excellent mechanical properties, chemical resistance, and far-infrared radiation [4,5,6,7]. Ceramic powder, which can emit far-infrared radiation ranging from 6 to 14 μm, plays an important role in the formation and growth of biological systems [8,9,10]. Far-infrared radiation can penetrate biological tissue and can have a strong rotational and vibrational energy effect at the molecular level. Since the absorption of far-infrared radiation can cause resonance within biological cells, far-infrared radiation ceramic (cFIR) composites can transfer energy to organisms and can result in a wide variety of biological effects [11,12]. Long-wavelength infrared radiation stimulates the activation of molecules (such as water) that are responsible for various biological reactions in the human body [13]. Ceramic powders with far-infrared emissivity have been applied to the skin in an effort to enhance blood flow [14]. Composites formed by embedding nanoscale Ge and SiO_2_ particles within polyvinyl alcohol nanofibers were observed to exhibit antimicrobial properties due to the emission of far-infrared radiation and can be used for biomedical applications [15].

Other potential applications for cFIR materials include textile fabrics, applied optics, coatings, and nanomembranes [16,17,18]. These materials can emit the energy absorbed from the environment into far-infrared rays. Furthermore, polymer composite materials based on cFIR have been proposed for various applications because they are likely to exhibit unique properties and environmental benefits [19]. Therefore, cFIR materials have attracted the attention of researchers owing to their unique properties [20,21,22].

**Figure 1:**
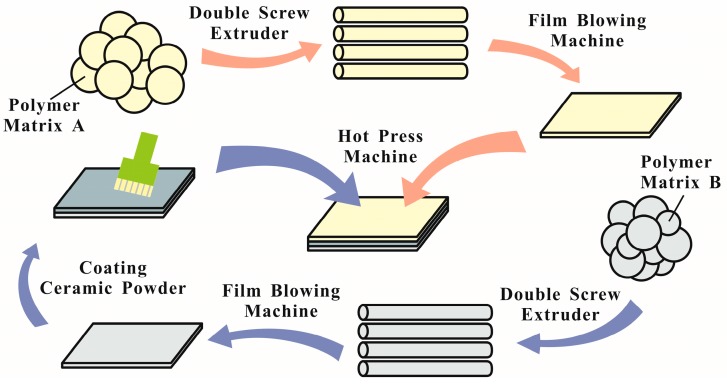
Detailed manufacture of sandwich-structured composites.

More investigations are needed to identify the real-world applications of cFIR composite films. This work characterizes the effect of ceramic powder on the far-infrared emissivity of polymer composites. Three different types of cFIR composite films were fabricated using a sandwich structure process (shown in Figure 1) that can enhance breakdown strength [23]. The cFIR films also show significant potential as packaging materials, as they can improve the shelf life of fruits owing to their biological effects. Strawberries have a high nutritional value but possess a short storage life. Hence, for the first time, we used far-infrared composite materials to package strawberries, with the aim of prolonging their shelf life and preserving their nutritional value.

## 2. Results and Discussion

### 2.1. Mineralogical Analysis of Ceramic Powder

**Figure 2:**
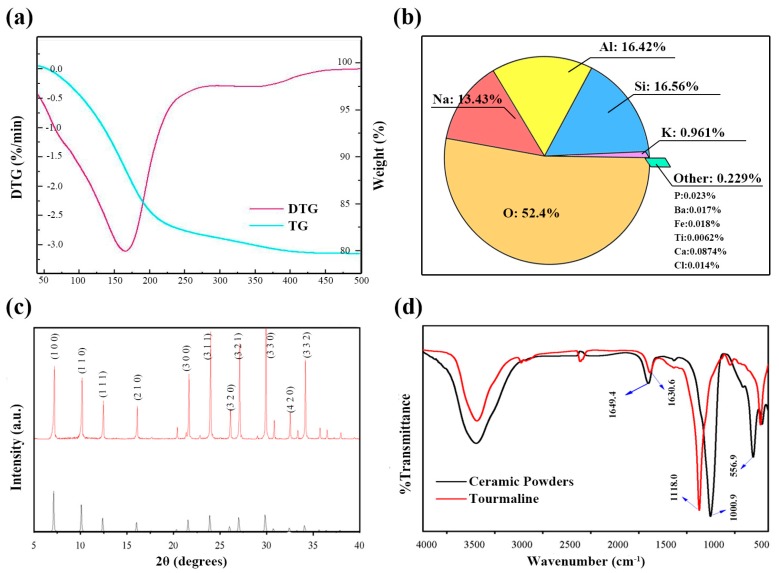
Mineralogical characterization of the ceramic powder: (**a**) TG and DTG curve; (**b**) major and minor elements (except for elements C and H); (**c**) XRD pattern; (**d**) FTIR spectra.

Figure 2a shows the TG-differential TG (TG-DTG) curves of the ceramic powder in the temperature range of 25–800 °C. In terms of the DTG curve, there are two endothermic peaks between 150 and 450 °C [24]. The endothermic peak near 160 °C indicates the dehydration reaction of hydrated ceramic. The peak at 360 °C is related to the decomposition and dehydration of inorganic salts. In the TG curve, there is a clear trend of decreasing mass between the two peaks, with a mass loss of 19.77%. Eventually, the mass of the sample stabilized at 450 °C, and the mass kept constant at 800 °C (not shown here), which may indicate the finish of sintering within this temperature range [25]. Since the emittance of a ceramic powder is dependent on its physical structure and mineralogical composition, this work investigated the spectral physical structure and the chemical composition [26,27]. The result of the mineralogical analysis via XRF in Figure 2b shows that the ceramic powder consisted of five major elements (O, Na, Al, K, and Si, Figure 2b) and seven minor elements (Ca, S, Cl, P, Ti, Fe, and Ba, Figure 2b). Oxygen had the highest concentration at 52.4% and titanium had the lowest concentration at 0.0062%.

As shown in Figure 2c, the X-ray diffractometry (XRD) pattern demonstrated the presence of alumina, titanium dioxide, and silicon dioxide in the ceramic powder, all of which were active in the far-infrared region. This result is in accord with a recent study indicating that the components of ceramic powder consist of natural zeolite, sodium aluminum silicate, and other phases [28]. The results obtained from FTIR are shown in Figure 2d. The high absorption capability will lead to a high emissive ability. The peaks of the infrared vibration bonds at 1649.4, 1000.9, and 556.9 cm^−1^ can be determined as C–O, Si–O, and Al–O, respectively. These bonds are identical to those in the spectrum of tourmaline (recognized as a natural mineral with strong infrared emission properties) in the 6–15 μm wavelength range [29,30,31,32]. In combination, these results indicate that ceramic powder is far-infrared active.

### 2.2. Properties of cFIR Composite Films

#### 2.2.1. Morphological Properties

**Figure 3:**
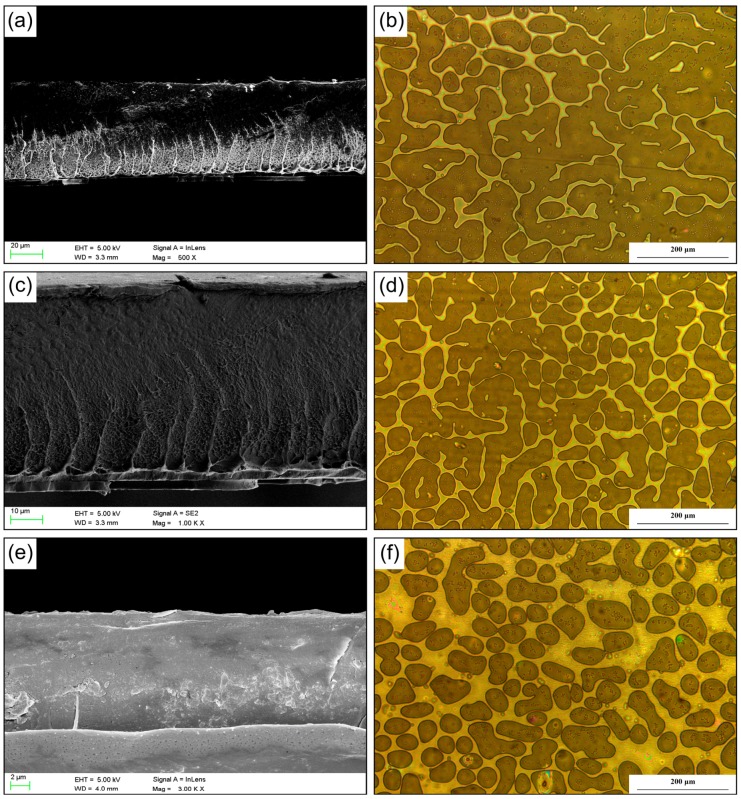
SEM and optical microscope images with different magnifications: (**a**,**b**) NY15/cFIR/PE60; (**c**,**d**) NY15/cFIR/PE40; (**e**,**f**) OPP38/cFIR/CPP40.

In order to study the dispersion behavior of cFIR in different films, optical microscopy and SEM were utilized at top and left views, respectively. Figure 3 shows the images of the NY15/cFIR/PE60, NY15/cFIR/PE40, and OPP38/cFIR/CPP40 films at different magnifications. A well-proportioned dispersion was observed in the different films. Perfect interfacial adhesion existed in different films without any aggregation of ceramic powder. A noticeable difference between NY/cFIR/PE and OPP/cFIR/CPP films was that the area of the ceramic powder in the NY/cFIR/PE film was larger than that in the OPP/cFIR/CPP film.

As a result, the sandwiched structure may have eliminated the stress concentration between the ceramic powder and the polymer matrix, which could have caused a sharp drop in the mechanical performance of the composite films. The morphologies of the samples indicate that the ceramic powder was evenly dispersed between the two films. Since a film must have uniform morphology to ensure optimal performance, this microstructure can lead to a well-proportioned distribution of far-infrared rays and guarantee accuracy in emissivity. On the other hand, a few specks can be seen in Figure 3, attributed to the separation and presence of solid impurities, which had no effect on the infrared emission performance.

#### 2.2.2. Mechanical Properties

Table 1 shows an overview of the mechanical properties of the composite film in the *x* and *y* directions including Young’s modulus, length at break, tensile stress, strength at break, and energy at break. The significant difference between the two directions can be clearly seen based on these five parameters.

Young’s modulus is a basic mechanical parameter that characterizes the elastic deformation properties of a material [33]. A composite with a large Young’s modulus is less prone to deformation, which means it will have better elasticity. According to Duncan’s variance analysis, the elastic deformation properties of the cFIR films were not significantly different from those of the control samples that lacked ceramic powder. The elastic deformation along the *y*-axis was larger than that along the *x*-axis. Comparing the three types of cFIR films, the elastic deformation of the NY/PE film was twice as large as that of the OPP/CPP film along the *x*-axis. However, the elastic deformation of the OPP/CPP film is five times larger than that of the NY/PE film along the *y*-axis. As can be seen in Table 1, the OPP/CPP film exhibited a more significant ability to stretch in the *y*-direction, with Young’s moduli of 268.05 and 330.17 MPa, compared to 17.11 and 15.24 MPa in the *x*-direction. Similarly, the elongation until break was also different in the two directions. The results, shown in Table 1, indicate that the tensile deformation in the *x*-axis is larger than that in the *y*-axis. The tensile deformation of the OPP/CPP film is larger than that of the NY/PE film in the *x*-axis.

It can be seen from Table 1 that, between the two axes, the Young’s modulus and elongation are significantly different, but the differences in tensile stress, strength at break, and energy at break in these two directions are relatively smaller. There are also significant differences among different composite materials. Table 1 shows that the three parameters of the OPP/CPP film are larger than those of the NY/PE film in both directions. The most striking observation from the data comparison was that most of the mechanical parameters of the cFIR films were nearly equivalent to those of the control films. (*p* ≥ 0.05). These results indicate that the mechanical properties of the cFIR composites are not significantly influenced (*p* ≥ 0.05) by the addition of ceramic powder.

The values of the mechanical parameters in the *y*-direction between the OPP38/CPP40 and OPP38/cFIR/CPP40 composites are significantly different (*p* < 0.05). The Young’s modulus values are 268.05 and 330.17 MPa. The tensile stress values are 13.01 and 11.86 MPa. The strength at break values are 152.24 and 138.79 N/tex, and the energy at break values are 1.77 and 1.13 J, respectively. This is likely due to the poor interfacial adhesion in the OPP38/cFIR/CPP40 sample, which can lead to stress concentration at the interface. However, there was no significant difference between the length at break values (*p* = 0.476 ≥ 0.05), which are 15.73 and 11.40 mm for the two samples.

#### 2.2.3. DMA and Far-Infrared Emissivity Properties

**Figure 4:**
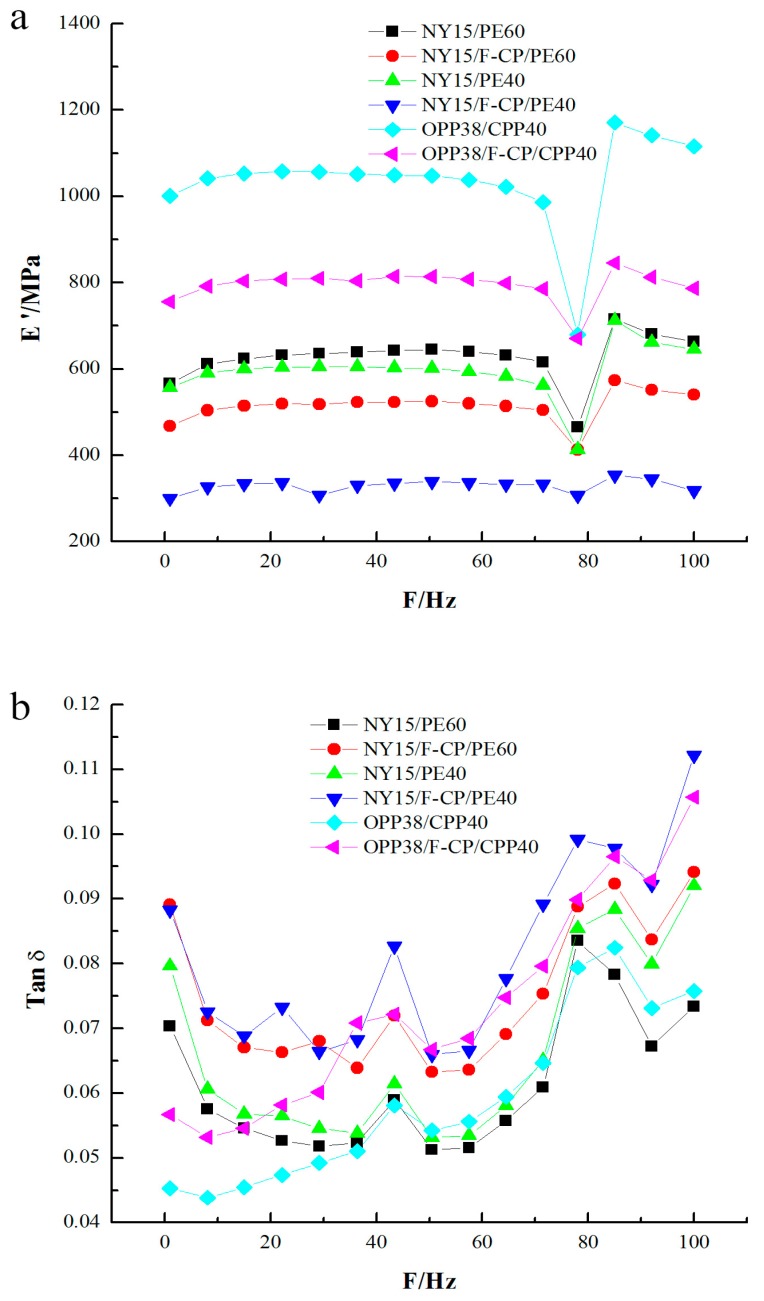
(**a**) Dynamical storage modulus; (**b**) tangent of loss angle of six composite films.

Figure 4a shows the dynamical storage modulus, and Figure 4b represents the tangent of the loss angle of various composite films as a function of the test frequency. It can be seen in Figure 4a that the addition of ceramic powder reduced the storage modulus of the composite material and reduced the stiffness, resulting in a decreased damping and shock absorption performance. Taking the NY15/cFIR/PE60 and NY15/cFIR/PE40 films as examples, Figure 4a shows that, with a decrease in the thickness of the cFIR composite films, the storage modulus and the stiffness are reduced accordingly. This is consistent with previous experience in practical applications. As can be seen from Figure 4a, the storage modulus of the OPP/CPP film is bigger than that of the NY/PE film. The storage moduli of these composite films have a linear relationship in the low-frequency region (0–70 Hz) for all samples. Figure 4a shows a significant fluctuation in the high-frequency region (70–100 Hz), and the linear relationship is no longer valid. At 80 Hz, the storage modulus is gradually reduced. There is a peak at a frequency above 80 Hz, and the damping properties of the composite at this point are minimal. Therefore, 80 Hz may be a critical point for the damping properties of these composite films, showing the best damping characteristics due to the fact that the films can maximally convert kinetic energy into heat loss at this frequency.

As can be seen in Figure 4b, there are several peaks in the tangent of the loss angle at 1, 40, and 80 Hz. A higher peak implies better impact resistance. With the addition of the ceramic powder, the stiffness of the material decreased but the anti-damping performance improved. When the load frequency exceeded 80 Hz, the storage modulus increased and the loss angle decreased. High-frequency embrittlement of the film was caused by the high storage modulus, and the working frequency of the composites was below 100 Hz.

The values of the far-infrared emissivity (ε) of the films prepared in this study (NY15/cFIR/PE60, NY15/cFIR/PE40, and OPP38/cFIR/CPP40) are listed in Table 2. The values for the control samples (NY15/PE60, NY15/PE40, and OPP38/CPP40) are also listed for comparison. The far-infrared emissivity of the composites were determined over the wavelength range of 5–14 µm at 25 °C using an infrared radiometer. The emissivity was measured as a relative value based on the assumption that the emissivity of a black body is 1. A black body with ε = 1 can convert almost all heat energy to electromagnetic energy. By adding cFIR, the emissivity of the polymers increased from 0.5 (control group) to 0.8, which is beyond the emissivity limitation of commercial products currently available [11]. Therefore, the addition of cFIR improved the far-infrared emissivity of the composites. cFIR composite films can efficiently transform energy from sunlight or other sources in the environment to far-infrared radiation. The generated radiation can then be re-emitted to the surroundings, similar to other far-infrared emitting materials [34].

### 2.3. Application for the Preservation of Strawberry

The strawberries were packaged by composite films to evaluate its application to fruit storage. As can be seen from Table 2, the rotting rate values on the 5th day are 16.7%, 17.5%, and 19.2% and 58.3%, 62.5%, and 67.3% for the cFIR composite and control samples, respectively. Additionally, weight loss rate values are 1.1%, 1.3%, and 1.4% in the cFIR composite films group and 2.0%, 2.2%, and 2.5% in the control group. Hence, the composite films with ceramic powder have a remarkable effect on the quality maintenance of strawberries and can effectively extend their shelf life. This was probably because the far-infrared emission property of the composite materials influenced cell metabolism and promoted the recovery of fatigued cells [35]. Possible mechanisms of this observed positive effect include the stimulation of water molecules and metabolism activation.

## 3. Materials and Methods

### 3.1. The Preparation of cFIR Composite Films

The far-infrared radiation ceramic powder (cFIR) used in this study was purchased and processed by the Zhuhai Deshen Environment-Friendly Packaging Co., Ltd., Zhuhai, China. Virgin polyester pellets of Nylon, polythene, casting polypropylene, and oriented polypropylene were fed into double screw extruder at a controlled rotational speed. The thermal profile used was different according to different pellets. Then, they were made into films with a film blowing machine. The ceramic powder was coated onto polythene and casting polypropylene films using a small brush. At last, different films were pounded into composites with a hot press machine.

The ceramic powder was dispersed between a nylon film and a polythene film (NY/cFIR/PE) and between an oriented polypropylene film and a cast polypropylene film (OPP/cFIR/CPP). The sandwich structures had known concentrations of ceramic powder between the two films. Three different composite films were produced—NY15/cFIR/PE60, NY15/cFIR/PE40, and OPP38/cFIR/CPP40—having total thicknesses of 0.75 mm, 0.55 mm, and 0.78 mm, respectively. It should be noted that the number next to the polymer’s short-form name defines the thickness of the polymer layer in the composite. cFIR refers to the far-infrared radiation ceramic powder that was dispersed between the two polymer layers. The control group without the ceramic powder (NY15/PE60, NY15/PE40, and OPP38/CPP40) were prepared for comparison. After cooling at 25 °C, the composite films were cut to have a length of 220 mm and a width of 170 mm.

### 3.2. Ceramic Powder Characterization

X-ray fluorescence spectrometry (XRF, Bruker AXS, Karlsruhe, Germany) and X-ray diffractometry (XRD-7000, Shimadzu, Japan) were used to characterize the inorganic compositions of the ceramic powder. XRD scans were obtained using Cu Kα radiation over the scanning range from 5° to 40°. Crystal phase were fitted using powder cell software (MDI Jade 5.0) [20]. The infrared vibrational bonds were analyzed using Fourier transform infrared spectroscopy (1800, PerkinElmer, Waltham, MA, USA) over the 400–4000 cm^−1^ range using a potassium bromide pellet. Thermogravimetric (TG) analysis was carried out with a TG analyzer (TG 209 F1, Netzsch, Bavaria, Germany), where 10 mg of sample was placed in a platinum crucible and heated from 25 to 800 °C at a rate of 20 °C·min^−1^ under a nitrogen flow of 50 mL·min^−1^.

### 3.3. Characterization of cFIR Composite Films

The morphology of the far-infrared composite films were characterized by optical microscopy (DM 4000, Leica, Solms, Germany) and field emission scanning electron microscope (Zeiss SIGMA, Heidenheim, Germany). The mechanical properties in the transverse and longitudinal directions were characterized using a tension tester (Model 3365, Instron, Boston, MA, USA) operated at a crosshead speed of 30 mm·min^−1^ under ambient conditions of 25 °C and 75% humidity. Dynamic mechanical analysis (TA Instruments, New Castle, PA, USA) was used to measure the damping property at 1–100 Hz in the tensile mode.

An infrared radiometer (EMS302M, Hede, Taiwan) was applied to analyze the emissivity of the composite films. Strawberries were obtained from a local commercial orchard in Wu’han, China, and the fruits were delivered to the laboratory immediately after being harvested. The specimens were carefully selected for uniform size and color; only specimens free of mechanical injury and visual defects were used. The specimens were randomly divided into two groups for the experiments. For rotting rate and weight loss rate, the specimens from each replicate (10 × 3 packaging) were used. Specimens were graded in five-levels and estimated according to the method of Li [36].

### 3.4. Statistical Analysis

The composite films were studied using a randomized design. The experimental data were presented as the mean ± standard deviation (SD) for three replicate samples. Significant differences between the mean values were determined using Duncan’s multiple range test (*p* < 0.05). Statistical analysis was performed using the software SPSS 13.0 (SPSS Inc., Chicago, IL, USA).

## 4. Conclusions

The main goal of this study was to prepare high emissivity composite films based on far-infrared ceramic powder. It was confirmed that ceramic powder has the capability of emitting far-infrared radiation. The most significant finding was that the mechanical properties of the cFIR composite film were not significantly influenced (*p* ≥ 0.05) by the addition of the ceramic powder, which may be attributed to the sandwiched structure. However, the addition of the ceramic powder decreased the damping and shock absorption performance of the composite films. The cFIR films showed high efficiency in transforming and emitting energy. In terms of cFIR film applications, our detailed experimental results suggested that such films could prolong the shelf life of strawberries owing to its far-infrared emission properties.

This study strengthens the idea that cFIR composite films are novel and promising materials that can be used in far-infrared applications. Further research should be undertaken to explore the potential applications of these films, such as the preservation of other fruits and vegetables, improvement in water quality, and physical therapy of the human body. Meanwhile, the preservation mechanism should be investigated in detail in future work.

## Figures and Tables

**Table 1:** Mechanical properties of far-infrared composite materials along the *x*-axis and the *y*-axis.

	Young’s Modulus (MPa)	Elongation at Break (mm)	Tensile Tress (MPa)	Strength at Break (N/tex)	Energy at Break (J)
	Mean (±SD)	Mean (±SD)	Mean (±SD)	Mean (±SD)	Mean (±SD)
***x*-Axis**					
NY15/PE60	35.02 ^e,f^ ± 3.13	47.44 ^b^ ± 4.58	5.88 ^d,e,f^ ± 0.45	48.53 ^h^ ± 3.71	1.60 ^b,c^ ± 0.23
NY15/cFIR/PE60	39.82 ^e^ ± 3.45	43.48 ^b^ ± 5.87	5.55 ^e,f^ ± 0.43	45.76 ^h^ ± 3.51	1.40 ^c,d^ ± 0.24
NY15/PE40	28.25 ^f^ ± 2.68	48.26 ^b^ ± 3.53	4.54 ^g^ ± 0.46	51.07 ^g,h^ ± 5.20	1.83 ^b^ ± 0.16
NY15/cFIR/PE40	30.28 ^f^ ± 2.95	45.24 ^b^ ± 4.86	4.38 ^g^ ± 0.39	49.29 ^g,h^ ± 4.43	1.59 ^b,c^ ± 0.27
OPP38/CPP40	17.11 ^g^ ± 1.65	72.53 ^a^ ± 6.44	7.05 ^c,d^ ± 0.31	82.44 ^c^ ± 3.67	4.31 ^a^ ± 0.54
OPP38/cFIR/CPP40	15.24 ^g^ ± 1.37	76.56 ^a^ ± 5.78	6.48 ^c,d,e^ ± 0.77	75.76 ^c,d^ ± 9.07	4.41 ^a^ ± 0.52
***y*-Axis**					
NY15/PE60	64.30 ^c^ ± 2.68	25.30 ^d^ ± 2.52	6.44 ^c,d,e^ ± 1.33	53.10 ^f,g,h^ ± 10.94	0.91 ^e^ ± 0.16
NY15/cFIR/PE60	60.45 ^c^ ± 2.99	29.51 ^c,d^ ± 3.65	7.41 ^c^ ± 0.79	61.13 ^e,f,g^ ± 6.49	1.15d ^e^ ± 0.25
NY15/PE40	48.01 ^d^ ± 3.07	31.27 ^c^ ± 2.54	5.65 ^e,f^ ± 0.53	64.83 ^d,e,f^ ± 4.92	1.35 ^c,d^ ± 0.16
NY15/cFIR/PE40	51.56 ^d^ ± 4.03	30.75 ^c,d^ ± 4.49	6.10 ^d,e^ ± 0.69	68.59 ^d,e^ ± 7.81	1.38 ^c,d^ ± 0.32
OPP38/CPP40	268.05 ^b^ ± 10.84	15.73 ^e^ ± 1.61	13.01 ^a^ ± 1.72	152.24 ^a^ ± 20.08	1.77 ^b,c^ ± 0.26
OPP38/cFIR/CPP40	330.17 ^a^ ± 10.76	11.40 ^e^ ± 0.92	11.86 ^b^ ± 0.88	138.79 ^b^ ± 10.33	1.13 ^d,e^ ± 0.14

Means with different letters are significantly different by Duncan’s multiple range test. ^a–g^ means value indicate significant differences between various species.

**Table 2:** Emissivity of cFIR composite films and the effects of packaging on strawberry.

Composite Films	Far-Infrared Emissivity (ε)	Rotting Rate (%)	Weight Loss Rate (%)
NY15/PE60	0.512	58.3	2.0
NY15/cFIR/PE60	0.861	16.7	1.1
NY15/PE40	0.508	62.5	2.2
NY15/cFIR/PE40	0.860	17.5	1.3
OPP38/CPP40	0.522	67.3	2.5
OPP38/cFIR/CPP40	0.866	19.2	1.4
